# Chloroform and Sir James Y. Simpson

**Published:** 1870-02

**Authors:** Jacob Bigelow

**Affiliations:** Boston


### Chloroform and Sir James Y. Simpson.

BY JACOB BIGELOW, M. D., OF BOSTON.

In a recent copy which has reached us of the *Edinburgh Daily Review*, is contained an account of a meeting of the Town Council of Edinburgh, at which the freedom of the city was presented in a crimson velvet box, emblazoned with the city arms, to Sir James Y. Simpson, Bart., M. D., etc. etc. The account is accompanied with the speech of the Lord Provost and the reply of the eminent physician to whom this signal honor was tendered. In the address the Lord Provost says, "I will not dwell on what you have accomplished in medical science. I will only allude to your discovery—the greatest of all discoveries in modern times—the application of chloroform to the assuagement of human suffering."

No one will probably object to the proceeding of the municipal authorities of Edinburgh in conferring high honor on one of its citizens who has assisted in introducing into that city the results of an important discovery, and whose professional celebrity, like that of many predecessors, has attracted to his place of residence an influx of strangers, thereby greatly benefiting "the hotel keepers, merchants and others of the city," not including the various manufactories of chloroform in Great Britain, one of which, "located in Edinburgh, makes as many as 8000 doses a day." But many persons will think it a mistake in the adopter of a foreign discovery to ignore the source from which he derived it. Sir James Simpson, in a long and eloquent reply to the Lord Provost, while he complacently accepts the crown of borrowed plumes thus tendered to him, makes not the slightest allusion to the country from which they were plucked, in which country anæsthetic inhalation, with more agents than one, was established, vindicated and successfully practiced long before it was heard of in Edinburgh or any part of Europe.

It is not wonderful that in the designs of Providence medicinal agents should exist, capable of averting pain by the suspension of sensibility. But the wonder is that after mankind had borne pain ever since the creation of their race, any person should be found of sufficient courage and strength of conviction to put through the untried and formidable experiments necessary to decide whether life could continue, under the inhalation of a scarce respirable vapor, carried to such an extent as to destroy sensibility and produce apparent death. That man was not Sir James Y. Simpson.

The history of anæsthetic inhalation is well known. It began in this country, and was first used in the extraction of teeth, and afterwards in capital operations in the Mass. General Hospital, and in obstetrical practice. The attention of the civilized world was immediately drawn to the great American discovery. Every known varieties of ethers and of compounds containing the elements of ethers together with volatile substances, gases and vapors, were at once submitted to the test of experiment. It is possible that better agents than those now in use will hereafter be discovered, but for the last twenty years the anæsthetic practice seems to have settled mainly on two agents, viz., sulphuric ether, with which the discovery was made, and which has thus far shown itself to be the most safe and manageable, and chloroform, which is more portable and agreeable in its odor, but which experience has shown to be more frequently attended with danger in its use.—*Boston Med. Journal*.

